# Historical pursuits of the language pathway hypothesis of schizophrenia

**DOI:** 10.1038/s41537-021-00182-z

**Published:** 2021-11-09

**Authors:** Lynn E. DeLisi

**Affiliations:** 1 Department of Psychiatry, Cambridge Health Alliance, Cambridge, MA USA; 2 Harvard Medical School, Cambridge, MA USA

**Keywords:** Genetics of the nervous system, Biomarkers

**arising from** Du et al. *npj Schizophrenia* 10.1038/s41537-021-00141-8 (2021)

I read with great interest the publication recently in this journal by Du et al.^1^, examining language dysconnectivity early in the illness course of schizophrenia and relating its occurrence to genetic determinants of language. These authors found elevated functional connectivity between Broca’s area for speech production, the thalamus and temporal lobe in first-episode schizophrenia, but dysconnectivity between Broca’s area and the anterior cingulate early in the illness onset that was associated with high polygenic risk scores for schizophrenia calculated specifically from FOXP2 language-related genes. While these authors suggest that genes for language functioning are related to the dysconnectivity that is associated with early schizophrenia, and thus the development of schizophrenia itself, they fail to recognize the extensive genetic risk literature that implicates entirely different genes. They also failed to adequately acknowledge the extensive literature on language and the genetics of schizophrenia that proceeded their study, not discussing how their results coincide with what came before them,

This is not at all a new concept that complex human language, schizophrenia, and the genetics of both are somehow connected. In fact, prior to the ability to examine language pathways by fMRI, there were several investigators who focused on language disconnectivity as the core feature of schizophrenia, documenting the many characteristic peculiarities to speech (reviewed in^2^) Others, such as Timothy Crow, theorized about the origins of the underlying deficit and postulated that schizophrenia derives from the uniquely human capacity for complex language, and went further to suggest that an anomaly in the genetic determinants of human language is responsible for the development of schizophrenia^3,4^. Crow further suggested that delusions and hallucinations, manifest symptoms of schizophrenia, come about because of disturbances in the pathways for language production^5^. Elaine Chaika^6^, who attributed the language disturbances in schizophrenia to dysfunctional executive control or a deficit in either working or semantic memory, was well known for her work examining speech passages she taped of patients describing what they had seen in a movie about a child asking his mother for money to buy ice cream. Numerous investigators since Chaika, such as Manschreck et al.^7^, Elvevåg et al.^8^, Sommer et al.^9^ and Corcoran et al.^10^, more recently became interested in language and published new studies about its importance in schizophrenia early on, even in the prodromal stage.

Ultimately these data led us to further use thought and language stimuli in fMRI studies to examine anomalies in multiple brain pathways for producing language in schizophrenia (reviewed in refs. ^11,12^. It is thus always important when reading new publications to be cognizant of what came before them in order to place them in context. Indeed, a whole body of literature preceded the Du et al. paper examining various MRI measures of language pathway abnormalities in schizophrenia (e.g.^13–19^. The overall pattern of findings indicates reduced functional and structural laterality in regions for language functioning.

With regard to the genetic nature of these changes, while variations in genes that influence the development of language pathways have been long hypothesized to be underlying the genetic basis for schizophrenia, there is no current evidence that suggests that the genes selected by Du et al. confer elevated risk for schizophrenia (Schizophrenia Working Group of the Psychiatric Genomics Consortium^20^; https://www.med.unc.edu/pgc/pgc-workgroups/schizophrenia/), even in any of the rare variants that have been shown in families^21–23^. Thus, this specific analysis in the paper published by Du and colleagues needs to be read with caution and awaits replication.

The genetics of schizophrenia remains elusive and is likely to be highly heterogeneous. While on the one hand many people with schizophrenia may have inherited a large portion of common modest risk genes^18^, others may come from families where inheritance is stronger and clusters in several individuals with rare unique variants contributing to illness (e.g.^21,22^. It will be difficult to implicate particular genes for human language, as it is likely that human language was made possible by many genes coming together to be activated in a precisely controlled, timed and coordinated process longitudinally, from the early perinatal years through age 2 when words begin to form in sentences. Thus, it is highly unlikely that studies such as the one described by Du et al., will be able to uncover specific genetic determinants for this portion of brain development that puts people at high risk to develop schizophrenia. There are, however, MRI studies suggesting different patterns of change in both structural and functional MRI that are thought to be associated with the genetic risk for schizophrenia.
